# ORGANIZATIONAL CONTEXT AND QUALITY INDICATORS IN NURSING HOMES: A MICROSYSTEM LOOK

**DOI:** 10.1093/geroni/igac059.1240

**Published:** 2022-12-20

**Authors:** Yinfei Duan, Alba Iaconi, Yuting Song, Matthias Hoben, Leslie Hayduk, Peter Norton, Carole Estabrooks

**Affiliations:** University of Alberta, Edmonton, Alberta, Canada; University of Alberta, Edmonton, Alberta, Canada; Qingdao University, Qingdao, Shandong, China (People's Republic); University of Alberta, Edmonton, Alberta, Canada; University of Alberta, Edmonton, Alberta, Canada; University of Calgary, Calgary, Alberta, Canada; University of Alberta, Edmonton, Alberta, Canada

## Abstract

This cross-sectional quantitative sub-project assessed the association of organizational context (modifiable elements of work environments) with quality indicators (QIs) at the clinical microsystem (care unit) level. We used TREC data collected 09/2019-03/2020. The sample included 285 care units within 91 Western Canadian nursing homes. Outcomes included thirteen practice-sensitive QIs derived from the Minimum Data Set 2.0. Results from random-intercept logistic regression for each dichotomized QI showed that higher unit-aggregated scores on contextual elements as identified by the Alberta Context Tool, specifically care aide participation in decision-making (OR=3.7-8.4, p<.05), care aide perceived staffing (OR=2.6, p<.05) and time for completing tasks (OR=5.1-7.0, p<.05), and care aide rated unit-level leadership (OR=20.1, p<.05), were associated with a better unit-level performance on delirium symptoms, indwelling catheter use, behavioral symptoms, pain, and late-loss physical function. The findings suggest that targeting modifiable contextual elements is an important avenue for quality improvement interventions in nursing homes.

